# Screening of genes co-associated with osteoporosis and chronic HBV infection based on bioinformatics analysis and machine learning

**DOI:** 10.3389/fimmu.2024.1472354

**Published:** 2024-09-16

**Authors:** Jia Yang, Weiguang Yang, Yue Hu, Linjian Tong, Rui Liu, Lice Liu, Bei Jiang, Zhiming Sun

**Affiliations:** ^1^ Clinical College of Neurology, Neurosurgery and Neurorehabilitation, Tianjin Medical University, Tianjin, China; ^2^ Department of Cardiovascular Surgery, Tianjin Medical University General Hospital, Tianjin, China; ^3^ Clinical School of the Second People’s Hospital, Tianjin Medical University, Tianjin, China

**Keywords:** osteoporosis, HBV, bioinformatics, machine learning, disease typing, immune cell infiltration

## Abstract

**Objective:**

To identify HBV-related genes (HRGs) implicated in osteoporosis (OP) pathogenesis and develop a diagnostic model for early OP detection in chronic HBV infection (CBI) patients.

**Methods:**

Five public sequencing datasets were collected from the GEO database. Gene differential expression and LASSO analyses identified genes linked to OP and CBI. Machine learning algorithms (random forests, support vector machines, and gradient boosting machines) further filtered these genes. The best diagnostic model was chosen based on accuracy and Kappa values. A nomogram model based on HRGs was constructed and assessed for reliability. OP patients were divided into two chronic HBV-related clusters using non-negative matrix factorization. Differential gene expression analysis, Gene Ontology, and KEGG enrichment analyses explored the roles of these genes in OP progression, using ssGSEA and GSVA. Differences in immune cell infiltration between clusters and the correlation between HRGs and immune cells were examined using ssGSEA and the Pearson method.

**Results:**

Differential gene expression analysis of CBI and combined OP dataset identified 822 and 776 differentially expressed genes, respectively, with 43 genes intersecting. Following LASSO analysis and various machine learning recursive feature elimination algorithms, 16 HRGs were identified. The support vector machine emerged as the best predictive model based on accuracy and Kappa values, with AUC values of 0.92, 0.83, 0.74, and 0.7 for the training set, validation set, GSE7429, and GSE7158, respectively. The nomogram model exhibited AUC values of 0.91, 0.79, and 0.68 in the training set, GSE7429, and GSE7158, respectively. Non-negative matrix factorization divided OP patients into two clusters, revealing statistically significant differences in 11 types of immune cell infiltration between clusters. Finally, intersecting the HRGs obtained from LASSO analysis with the HRGs identified three genes.

**Conclusion:**

This study successfully identified HRGs and developed an efficient diagnostic model based on HRGs, demonstrating high accuracy and strong predictive performance across multiple datasets. This research not only offers new insights into the complex relationship between OP and CBI but also establishes a foundation for the development of early diagnostic and personalized treatment strategies for chronic HBV-related OP.

## Introduction

Hepatitis B virus (HBV) infection is a significant global public health issue, affecting millions of people’s health (1, 2). Chronic HBV infection (CBI) can lead to chronic liver diseases, including cirrhosis and hepatocellular carcinoma, severely impacting patients’ quality of life (3). Recent studies have found that CBI is not only associated with liver-related diseases but may also increase the risk of other comorbidities, including osteoporosis (OP) (4, 5). OP is a bone disease characterized by low bone density and deterioration of bone tissue structure, leading to fragile bones and an increased risk of fractures (6, 7). This disease is common in middle-aged and elderly people, especially postmenopausal women, but men and younger individuals are also at risk (8, 9). The development of OP is related to various factors, including genetics, diet, lifestyle, and the impact of chronic diseases (10).

“Hepatic osteodystrophy” is a common complication of chronic liver disease, characterized by increased bone resorption and decreased bone formation, leading to metabolic bone disease (11, 12). It has been reported that the incidence of OP in chronic liver disease ranges from 12% to 55%, with fracture risk reaching up to 40% (13). In patients with chronic hepatitis, OP is considered one of the most significant complications (14). The connection between CBI and OP is not yet fully understood, but research suggests that HBV may impact bone health through direct and indirect mechanisms. These mechanisms include the chronic inflammatory response induced by the viral infection, low serum levels of insulin-like growth factor I, the induction of tumor necrosis factor which inhibits bone formation, and the potential effects of HBV-related drug treatments on bone density (15–18).

Bioinformatics is an interdisciplinary scientific field that combines biology, computer science, and statistics (19). It utilizes computational technology and mathematical methods to process and analyze the massive amount of data produced in biological research, revealing the nature and mechanisms of biological phenomena (20). Bioinformatics is widely applied in areas such as genomics, transcriptomics, proteomics, and metabolomics (21). The rapid development of this discipline has been facilitated by high-throughput technologies, such as gene chips, high-throughput sequencing, and mass spectrometry analysis (22). These advanced technologies generate a vast amount of biological data, which require in-depth analysis with bioinformatics tools. Research topics include data mining, sequence alignment, protein structure prediction, and biological network analysis (23). These methods help to unearth valuable information from the data, such as gene function, metabolic pathways, and protein interactions, which are crucial biological questions (24). Machine learning (ML), a branch of computer science, provides machines with the ability to learn autonomously. Machine learning algorithms are widely used in bioinformatics for prediction, classification, and feature selection tasks, and their application in the field of bioinformatics has become an important force driving biological research and medical development (25). By analyzing clinical data, medical images, and transcriptome data, machine learning can help doctors diagnose diseases more accurately, classify diseases, and identify disease-specific gene expression patterns (26).

In this study, we collected four OP datasets and one HBV dataset from the Gene Expression Omnibus (GEO) database. Bioinformatics-based analysis and machine learning methods were used to screen for common pathogenic genes of HBV and OP, and predictive models were constructed. In addition, a column-line graph prediction model was constructed, and the prediction performance was evaluated using calibration curves, decision curve analysis (DCA), and clinical impact curves. OP patients were classified into cluster1 and cluster2 according to HBV-related genes (HRGs), and the mechanisms by which HRGs affect the occurrence and development of OP were further explored by enrichment analysis and immune cell infiltration analysis. Three core genes were finally identified, and samples from 10 patients with CBI and 10 patients with combined OP with CBI were collected from Tianjin Second People’s Hospital for molecular biology experiments.

## Methods

### Data collection and processing

This study collected five datasets from the Gene Expression Omnibus (GEO) database (https://www.ncbi.nlm.nih.gov/geo/) (27). The GSE83148 dataset includes liver tissue samples from 122 CBI patients and 6 healthy controls. GSE56815, GSE56814, GSE7429, and GSE7158 consist of peripheral blood mononuclear cell samples from OP patients and controls. The “sva” and “limma” packages in R software were used for data normalization and batch effect elimination (28, 29). The information for each dataset was shown in **Table 1**.

**Table 1:** Basic information of GEO datasets.

ID	Sample source	Number of cases	Number of controls
GSE83148	Liver	122	6
GSE56815	Peripheral blood mononuclear cell	40	40
GSE56814	Peripheral blood mononuclear cell	31	42
GSE7429	Peripheral blood mononuclear cell	10	10
GSE7158	Peripheral blood mononuclear cell	12	14

### Gene differential expression analysis

The R package “limma” was used to perform gene differential expression analysis on the combined datasets of GSE56815 and GSE56814, and separately on GSE83148. The criteria for inclusion of differentially expressed genes in the GSE83148 dataset were *P*<0.05 and LogFC|>1. For the combined dataset, the criterion for differentially expressed genes was *P*<0.05. Subsequently, an intersection of differentially expressed genes between the two datasets was taken.

### Screening genes by least absolute shrinkage selection operator analysis and machine learning

The “glmnet” package was used for LASSO analysis to further select genes within both the combined dataset and the GSE83148 dataset, choosing the lambda corresponding to the smallest Binomial Deviance as the optimal value (30). Within the combined dataset, the “caret” package was utilized to compare the effects of recursive feature elimination (RFE) between models such as Random Forest (RF), Support Vector Machine (SVM), and Gradient Boosting Machine (GBM), to determine the final HRGs. The combined dataset was divided into a training set and an internal validation set in an 0.8:0.2 ratio, followed by the construction of an SVM model through ten-fold cross-validation. The “caret” package automatically selects the optimal model. The “DALEX” package was used to interpret the SVM model and generate results for the distribution of residuals and gene importance ranking (31). The “pROC” package was employed to draw the ROC curve and calculate the Area Under the Curve (AUC) to assess the accuracy of the predictive model (32). The GSE7429 and GSE7158 datasets were used to validate the accuracy of the model.

### Construction and evaluation of the nomogram model

A nomogram model was constructed for the combined dataset using the Logistic regression method, and validated with the test datasets GSE7429 and GSE7158. The R software packages “rms” and “VRPM” were utilized to establish the nomogram model for OP risk assessment. In the nomogram, each of the HRGs is assigned a specific score; the individual scores of the 18 HRGs are summed to derive a total score. The risk of OP can be inferred based on the total score. The predictive capability of the nomogram model was evaluated using ROC curves, calibration curves, Decision Curve Analysis (DCA), and Clinical Impact Curves (33).

### Identification and functional enrichment analysis of HBV-related OP patient clusters

To explore the differences in HBV-related clusters among OP patients, we clustered OP patients using the non-negative matrix factorization (NMF) method (34). The clustering was executed using the R package “NMF”, applying the “brunet” algorithm over 50 iterations. Gene Ontology (GO) analysis, a common method for large-scale functional enrichment studies, encompasses biological processes (BP), molecular functions (MF), and cellular components (CC). The Kyoto Encyclopedia of Genes and Genomes (KEGG), a database widely used for biological pathway analysis (35), alongside the R package “clusterProfiler”, was employed for conducting and visualizing GO and KEGG enrichment analyses. Gene Set Variation Analysis (GSVA), facilitating gene set (pathway) level differential analysis (36), was performed using the R packages “GSVA” and “limma”. The R package “ggplot2” was utilized for visualization of the analysis results. GO, KEGG, and GSVA enrichment analyses were conducted to explore the differences in biological processes between aging-related clusters. GO and KEGG enrichment analyses were carried out using the Gene Set Enrichment Analysis (GSEA) method. Differentially expressed genes between clusters with |LogFC|>1 and *P*<0.05 were included in the analysis, and results with *P*<0.05 in the enrichment analyses were considered statistically significant. The GSVA enrichment analysis employed the single-sample gene set enrichment analysis (ssGSEA) method, which calculates pathway scores based on gene expression matrices (37). In the GSVA enrichment results, |t|>2 and *P*<0.05 were deemed statistically significant.

### Immune cell infiltration analysis

To identify differences in immune cell infiltration status among different HBV-related clusters, this study downloaded the commonly used immune cell-related gene set “LM22” from the literature (38). Using the R package “GSVA” and the ssGSEA algorithm, the immune cell infiltration scores of 71 OP samples in the GSE56815 dataset were evaluated to distinguish between the immune cell infiltration statuses of different clusters (39). Additionally, the R package “psych” was utilized to calculate the correlation between HRGs and 28 types of immune cells through Pearson correlation analysis.

### Patients’ samples collection and peripheral blood mononuclear cell isolation

In this study, 10 mL of fresh peripheral blood specimens were collected from 10 patients with CBI and 10 patients with CBI combined with OP at Tianjin Second People’s Hospital. PBMCs were prepared from peripheral blood specimens by density gradient centrifugation using Ficoll cushion. The clinical information of patients was shown in **Table 2**. The study was approved by the Ethics Committee of Tianjin Second People’s Hospital (No. [2018]15) and written informed consent was obtained from all participants.

**Table 2:** Patient demographics.

Characteristics	CBI (n=10)	CBI combined with OP (n=10)	*P* value
Age (years)	38.4 ± 4.8	63.2 ± 3.1	*<0.001*
Male sex	8 (80.0%)	7 (70.0%)	*0.605*
HBV DNA (Log10 IU/mL)	1.4 (1.2-1.7)	1.3 (1.1-1.4)	*0.912*
ALT(U/L)	17.9 ± 6.5	25.4 ± 11.9	*0.123*
AST(U/L)	27.4 ± 8.7	35.4 ± 12.1	*0.105*
GGT (U/L)	47.6 ± 9.3	45.5 ± 10.3	*0.109*

ALT, alanine aminotransferase; AST, aspartate aminotransferase; GGT, glutamyltransferase.

### The validation of the expression of hub genes between CBI and CBI combined with osteoporosis groups

Total RNA extraction was adopted using the Trizol reagent (Thermo Fisher Scientific, Darmstadt, Germany), followed by reverse transcription with a Reverse Transcription Kit (Takara Code No.RR 037A) following the instruction of the manufacturer. Real-time quantitative PCR (RT-qPCR) was performed by adopting a TaqMan PCR Kit (ThermoFisher). All reactions were conducted in duplicate, and the relative mRNA expression was calculated based on the 2^−ΔΔCt^ approach. Primer sequences are listed as follows: USP10-F, 5′-ATTGAGTTTGGTGTCGATGAAGT3′; USP10-R, 5′-GGAGCCATAGCTTGCTTCTTTAG3′; ECM1-F, 5′-GCTTCACGGCTACAGGACAG3′; ECM1-R, 5′-GAGGCTTCGGGATAGGGGT3′; ERAL1-F, 5′-TCAATCGGTGTTAAGAGTCTGGC3; ERAL1-R, 5′-TCCGTTGGAAGCCTAAGAGTG3′.

### Statistical analysis

R version 4.2.3 and GraphPad Prism version 9.0.2 (GraphPad Software Inc., San Diego, CA, USA) and SPSS 21.0 software (Chicago, IL, USA) were used for statistical analysis. The counting data were expressed as cases and percentages, and Chi-square test was used for comparison between groups. The statistical description of non-normal distribution data was expressed by median and quartile, and Mann–Whitney U test was used for comparison between groups. All tests were performed by two-tailed and *P* value of <0.05 was statistically significant.

## Results

### Gene differential expression analysis and screening of HRGs

**Figure 1:**
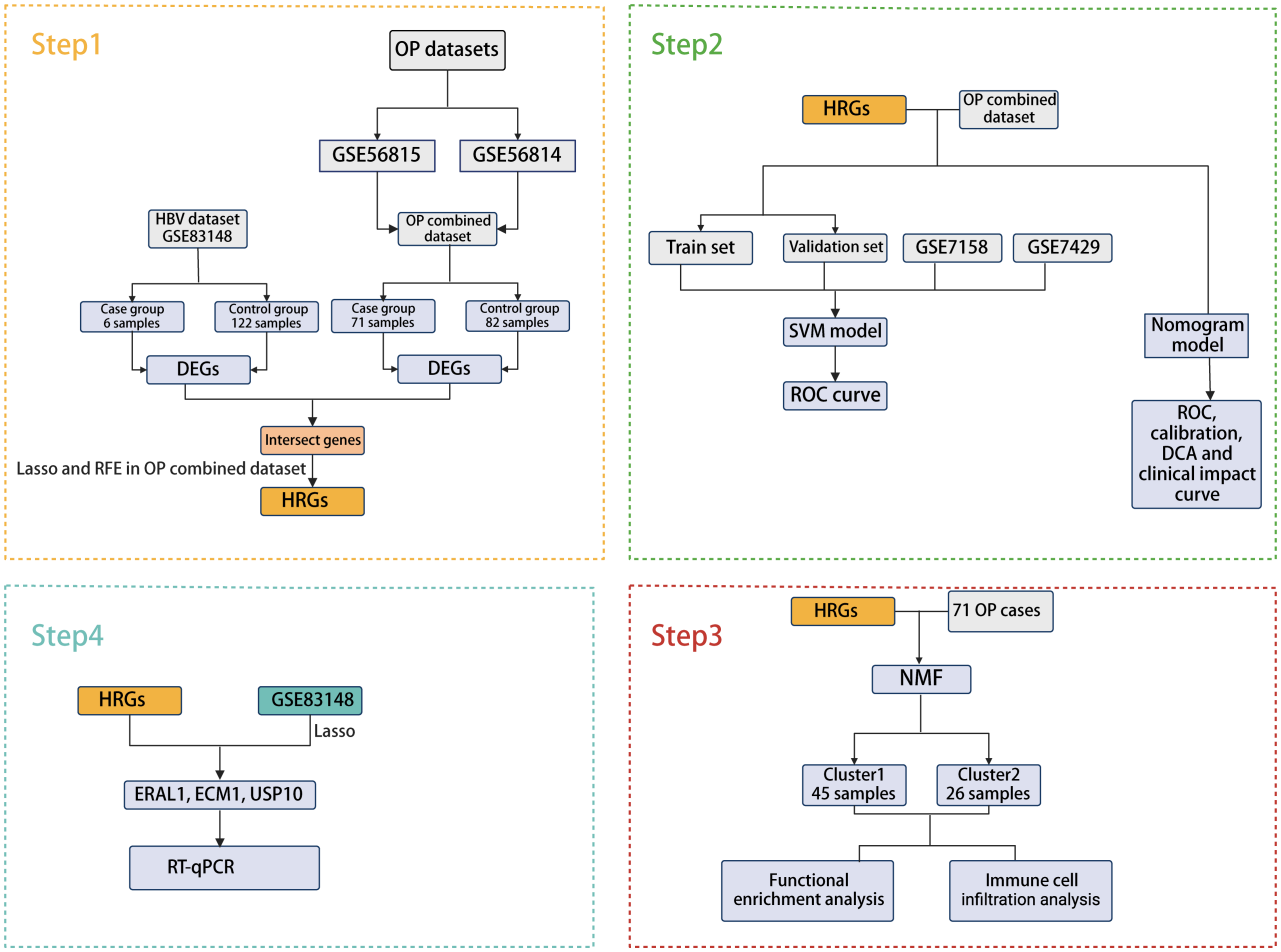
Flowchart of this study.

**Figure 2:**
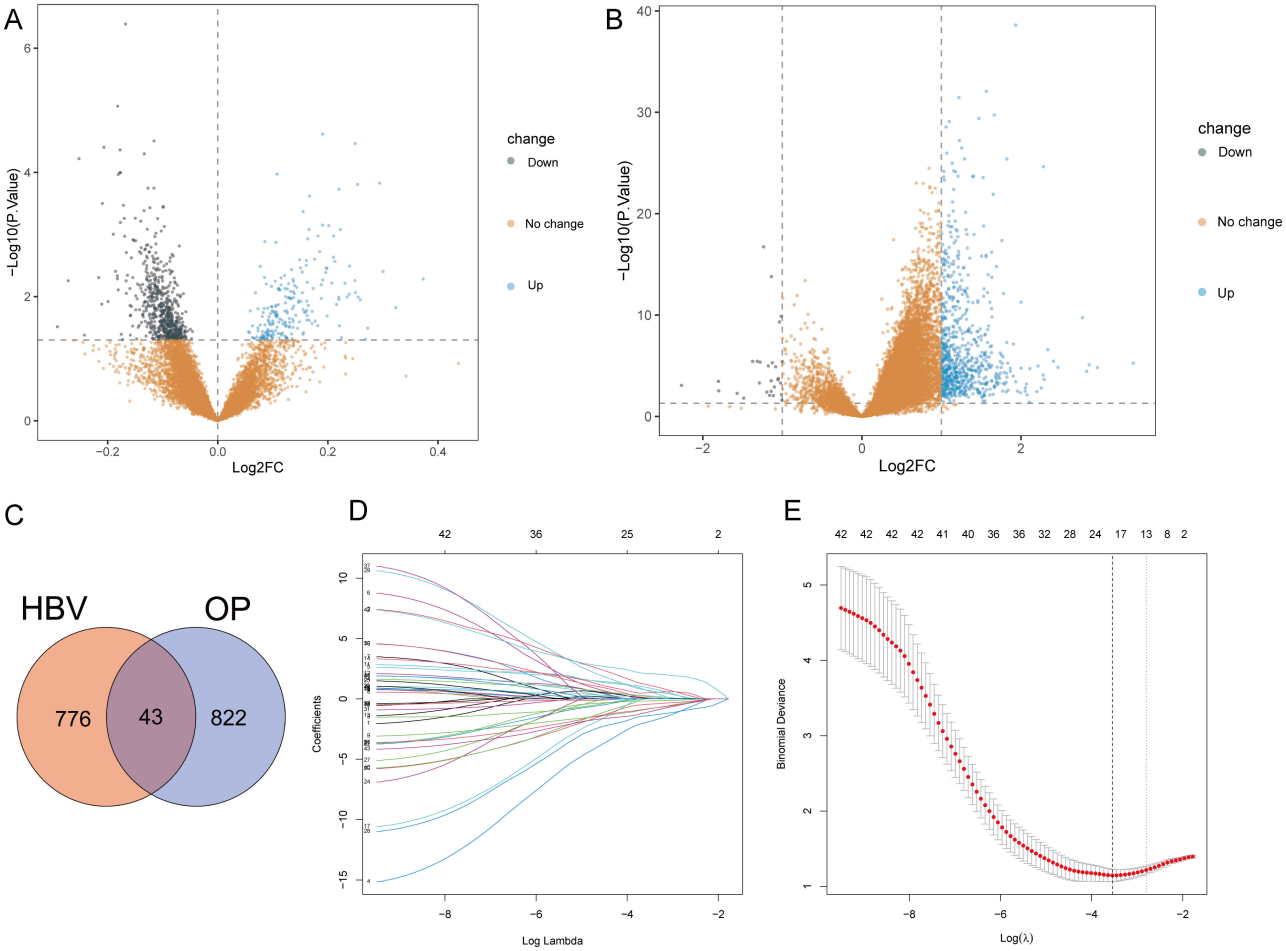
Gene differential expression analysis and Lasso analysis. **(A)** OP dataset. **(B)** GSE83148. **(C)** Intersection of DEGs from both datasets. **(D)** Coefficients of each gene as the penalty parameter lambda varies. Each line represents a gene. **(E)** Ten-fold cross-validation graph. The left dashed line represents the lambda value when the binomial deviation is minimal.

The bioinformatics analysis strategy is illustrated in **Figure 1**. The combined dataset of GSE56815 and GSE56814, including 71 OP patients and 82 healthy control samples, yielded 822 differentially expressed genes (DEGs) after gene differential expression analysis (**Figure 2A**). The GSE83148 dataset resulted in 776 DEGs after gene expression differential analysis (**Figure 2B**). An intersection of the two datasets revealed a total of 43 common genes (**Figure 2C**).

**Figure 3:**
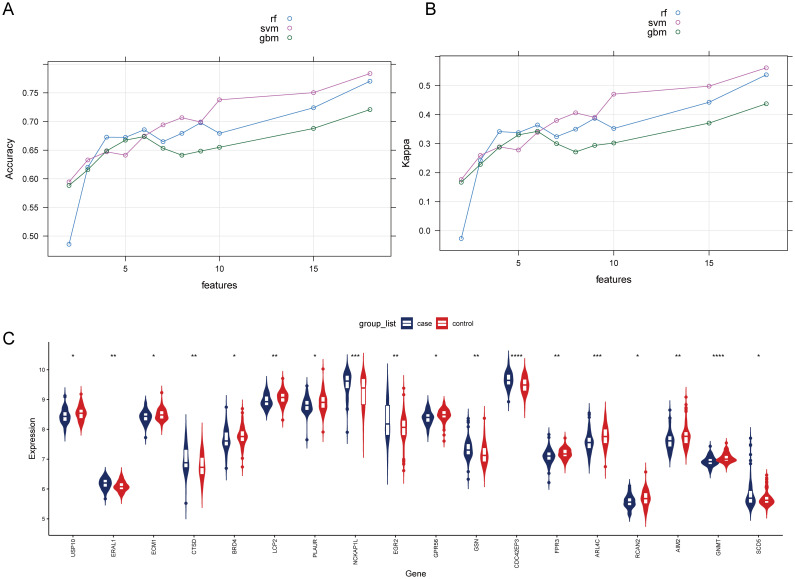
Comparison of machine learning models and expression of HRGs in the OP **(A)** Change in the accuracy of machine learning recursive feature elimination algorithms with the number of genes. **(B)** Change in the kappa of machine learning recursive feature elimination algorithms with the number of genes. **(C)** Expression of HRGs in the OP dataset. ^*^ *P*<0.05, ^**^ *P*<0.01, ^***^ *P*<0.001, ^****^ *P*<0.0001.

To select HRGs, LASSO analysis was performed on the combined dataset. **Figure 2D** illustrates the coefficient of each gene varying with lambda. At lambda = 0.02887027, where the Binomial Deviance was minimized, the number of HRGs was determined to be 18 (**Figure 2E**). Subsequently, this study aimed to further filter the HRGs using three machine learning methods: RF-RFE, SVM-RFE, and GBM-RFE, evaluating the predictive performance of each method through Accuracy and Kappa. The results indicated that SVM-RFE outperformed RF-RFE and GBM-RFE in both Accuracy and Kappa. Moreover, among the three machine learning methods, when the number of HRGs was 18, the SVM-RFE method exhibited the highest Accuracy and Kappa, at 0.78 and 0.56, respectively (**Figures 3A, B**). These 18 HRGs were identified as USP10, ERAL1, ECM1, CTSD, BRD4, LCP2, PLAUR, NCKAP1L, EGR2, GPR56, GSN, CDC42EP3, FPR3, ARL4C, RCAN2, AIM2, GNMT, and SCD5. **Figure 3C** illustrates the expression of HRGs in OP patients and healthy controls.

### Construction and evaluation of predictive models for OP of HRGs

**Figure 4:**
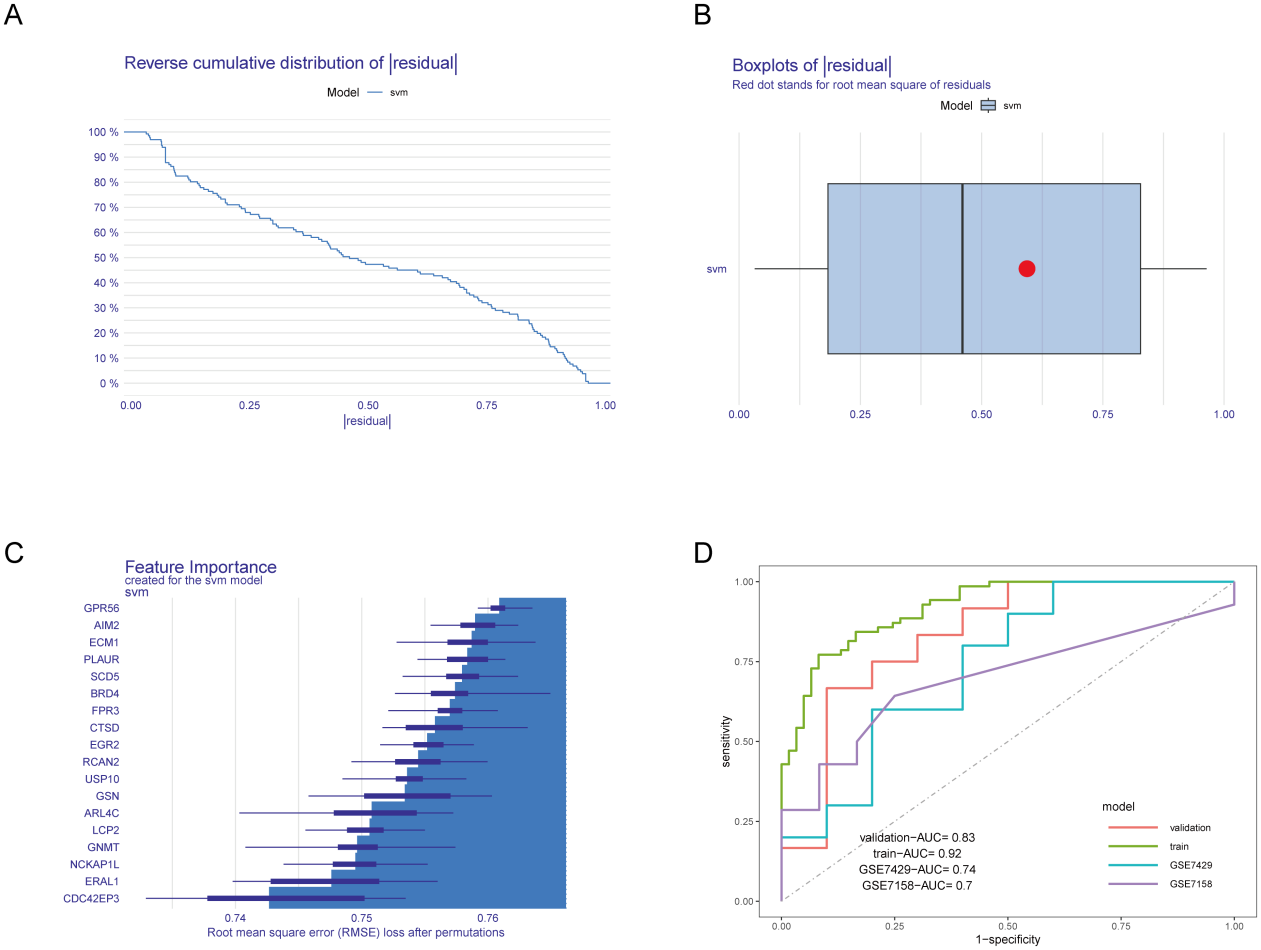
Construction and evaluation of the SVM model. **(A)** Residual inverse cumulative distribution curve. The X-axis represents the residual value. The Y-axis represents cumulative frequency. This means for any point on the graph, what frequency of samples have residuals greater than or equal to the value on the X-axis. **(B)** Residual box plot. The X-axis represents the residual value. The red dot represents the root mean square of residuals. **(C)** Importance of HRGs. The greater the RMSE loss, the more important the gene. **(D)** ROC curves of the SVM model in various datasets.

Next, we divided the combined dataset into a training set and a validation set in an 0.8:0.2 ratio and constructed an SVM model based on the 18 identified HRGs. We then analyzed and interpreted the distribution of residuals and the importance of features in the SVM model and evaluated the model’s performance through ROC curves. **Figures 4A, B** display the residual inverse cumulative distribution curve and box plot, respectively. **Figure 4C** shows the importance of HRGs in the SVM model, evaluated using Root Mean Square Error (RMSE) loss, with GPR56 ranked as the most important. Finally, we used the GSE7428 and GSE7158 datasets to validate and test the effectiveness of our predictive model. **Figure 4D** presents the AUC values of the ROC curve for the SVM model. The AUC values in the training set, validation set, GSE7429, and GSE7158 were 0.92, 0.83, 0.74, and 0.7, respectively. The results indicate that the SVM model constructed based on HRGs demonstrates good predictive ability and can be used to predict the risk of OP.

**Figure 5:**
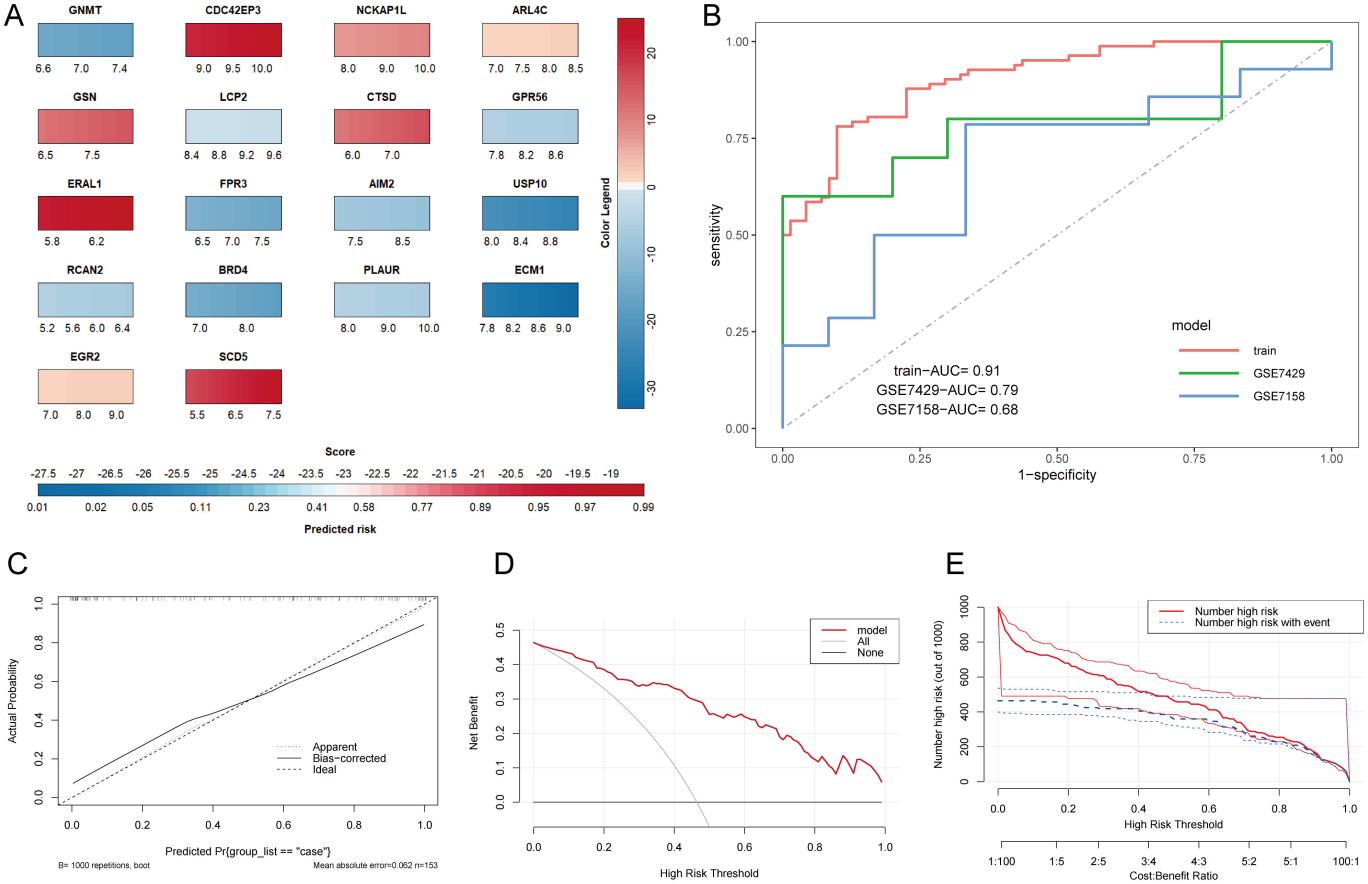
Construction and evaluation of the nomogram model. **(A)** Nomogram prediction model. The color legend on the right represents the score of each gene, adding up each gene’s score to get the Score below and the Predicted risk of OP. **(B)** ROC curves of the nomogram model in various datasets. **(C)** Calibration curve. The X-axis represents predicted probability, and the Y-axis represents actual outcomes. Apparent represents uncorrected model predictions. Bias-corrected represents model predictions after bootstrapping correction. Ideal represents absolute ideal model predictions. **(D)** DCA curve. The X-axis is the threshold probability for being judged as high-risk, and the Y-axis represents net benefit. All and None represent the extreme cases of all interventions and no interventions. **(E)** Clinical impact curve. The dual X-axis represents the threshold probability of being judged as high risk and the cost: benefit ratio. Number high risk represents the number of cases judged positive by the model, Number high risk with event represents the number of true positives.

To further evaluate the predictive performance of HRGs, we constructed a nomogram model based on the 18 HRGs using the combined dataset as the training set (**Figure 5A**). The ROC curve, calibration curve, Decision Curve Analysis (DCA) curve, and clinical impact curve were utilized to further assess the predictive performance of the nomogram. The nomogram model demonstrated an AUC of 0.91 in the training set, with AUCs of 0.79 and 0.68 in the GSE7429 and GSE7158 datasets, respectively (**Figure 5B**), indicating strong diagnostic value for OP. The calibration curve shows that the predicted performance of the constructed nomogram model aligns closely with the actual outcomes (**Figure 5C**). Similarly, the DCA curve illustrates the net benefit of the nomogram model across different risk thresholds, showing that decisions based on the nomogram model yield a net benefit compared to either intervening in all or none (**Figure 5D**). The clinical impact curve displays the estimated number of individuals identified as high risk by the model and the number of true positives across varying risk thresholds, aiding in assessing the model’s efficacy in identifying true cases (**Figure 5E**).

### Identification and functional enrichment analysis of CBI combined with OP patient clusters

**Figure 6:**
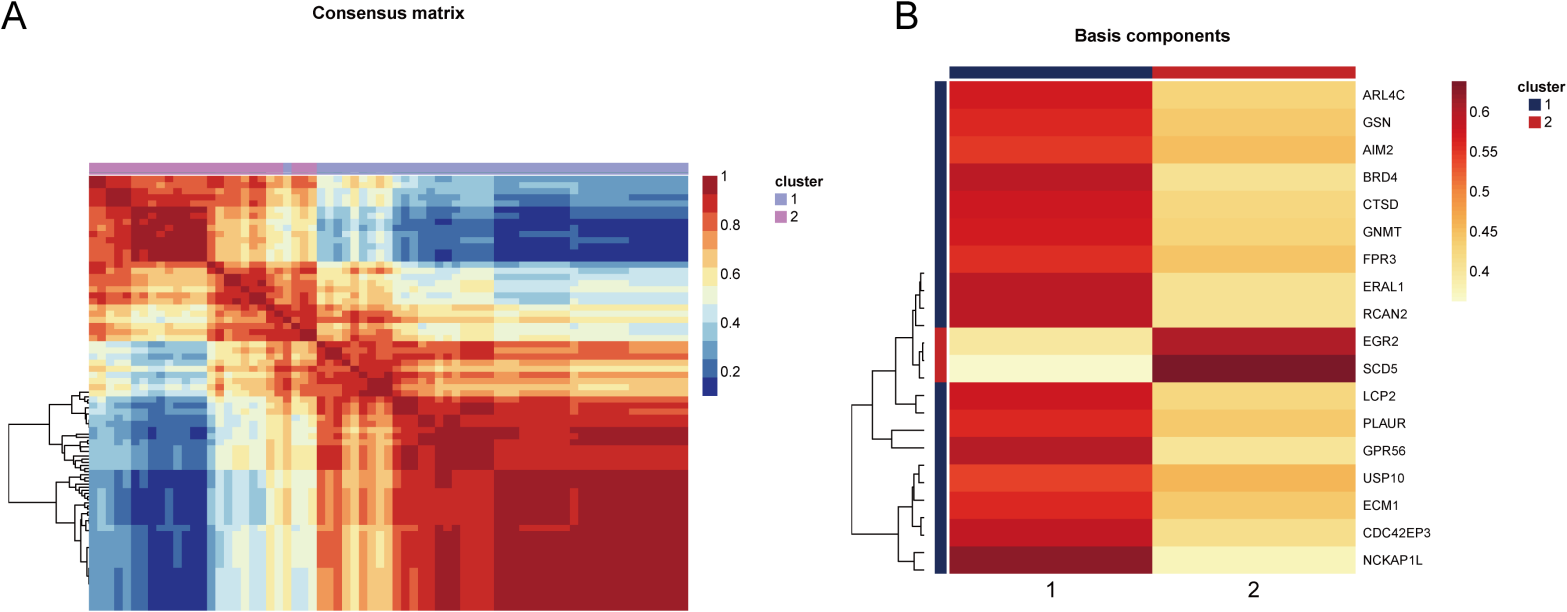
Heatmap showing the distinction between cluster1 and cluster2. **(A)** The consensus matrix of NMF clustering. **(B)** The co-clustering coefficient of HRGs.

**Figure 7:**
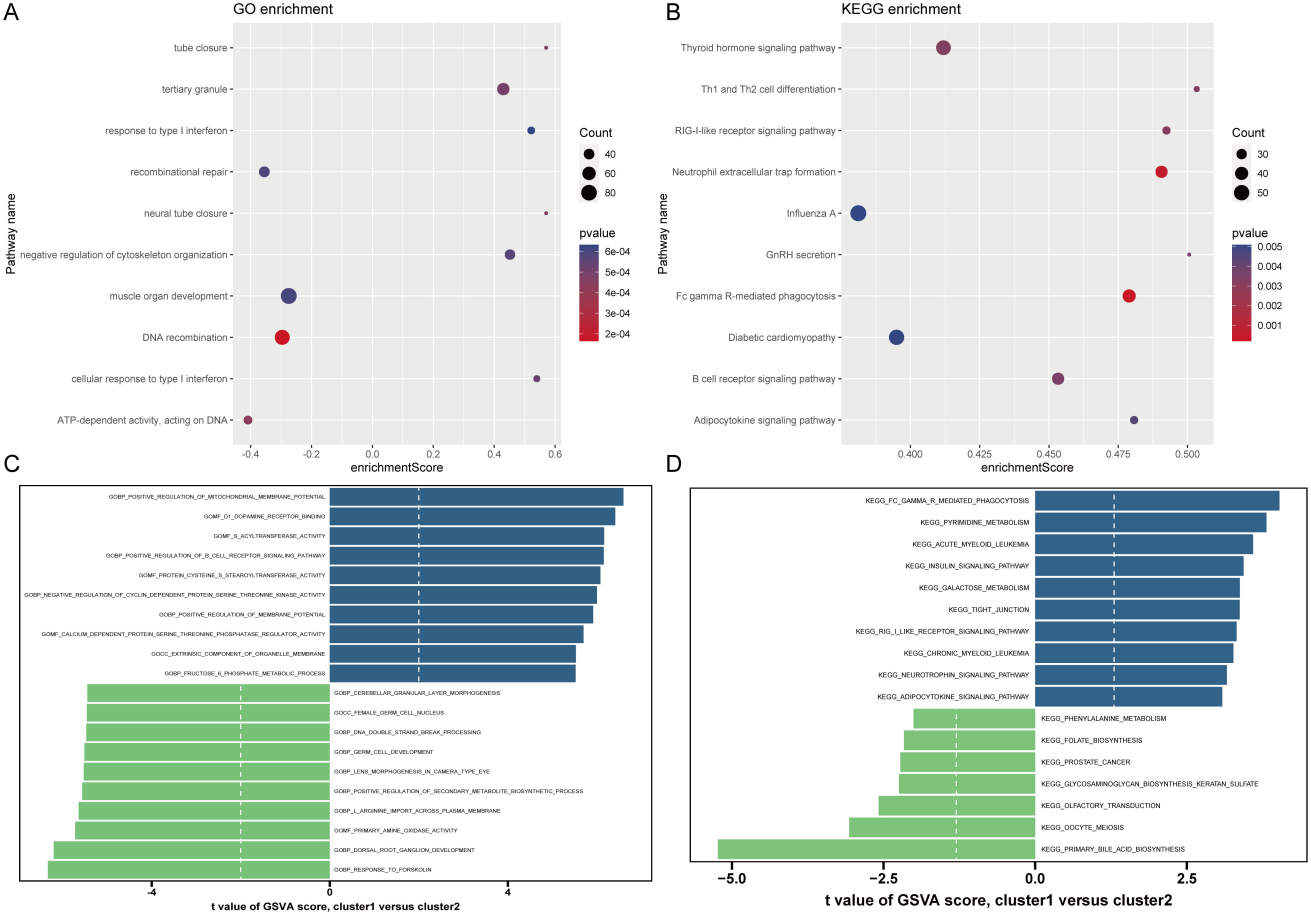
Results of GO and KEGG enrichment analysis. **(A, B)** GSEA results of GO and KEGG. The X-axis represents enrichment score. The Y-axis represents pathway names. Count represents the number of genes. **(C, D)** GSVA results of GO and KEGG. The X-axis is the t-value. The Y-axis represents pathway names.

Based on the 18 HRGs, patients in the combined dataset with OP were clustered using the Non-negative Matrix Factorization (NMF) method. A total of 71 OP patients were divided into cluster1 (N=45) and cluster2 (N=26). **Figures 6A, B** display the distinction between cluster1 and cluster2 through heatmaps. Subsequently, GO and KEGG enrichment analyses were performed between cluster1 and cluster2. **Figure 7A** and **Figure 7B** show the top 10 results of GSEA for GO and KEGG respectively, respectively. In the GSEA results for GO, compared to cluster2, cluster1 showed upregulation in response to type I interferon, tertiary granule, tube closure, negative regulation of cytoskeleton organization, neural tube closure, and cellular response to type I interferon, and downregulation in recombinational repair, muscle organ development, DNA recombination, and ATP-dependent activity acting on DNA. In the GSEA results for KEGG, cluster1, in comparison to cluster2, indicated upregulation in the Adipocytokine signaling pathway, B cell receptor signaling pathway, Diabetic cardiomyopathy, FcγR-mediated phagocytosis, GnRH secretion, Influenza A, Neutrophil extracellular trap formation, RIG-I-like receptor signaling pathway, Th1 and Th2 cell differentiation, Thyroid hormone signaling pathway. Similarly, **Figure 7C** shows the top ten results for upregulated and downregulated GO terms in GSVA for cluster1 relative to cluster2. **Figure 7D** presents the results for upregulated and downregulated KEGG pathways in GSVA, with 10 pathways being upregulated and only 7 downregulated. In other words, the upregulated results represent pathways primarily involved by cluster1, while the downregulated results represent pathways mainly involved by cluster2.

### Immune cell infiltration analysis

**Figure 8:**
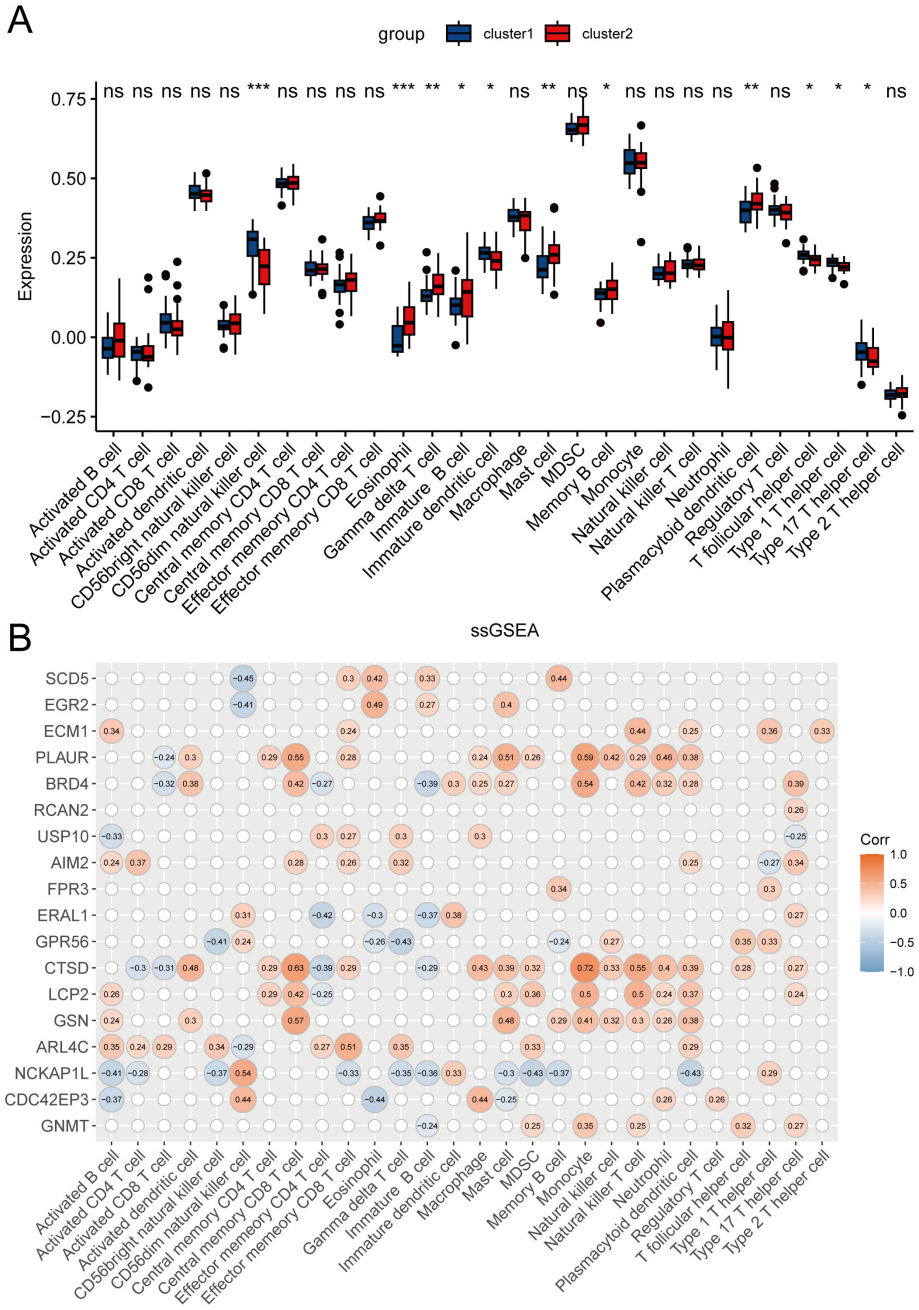
Immune cell infiltration analysis. **(A)** Differences in 28 types of immune cell infiltration between clusters evaluated by the ssGSEA algorithm. ^*^ *P* < 0.05, ^**^ *P* < 0.01, ^***^ *P* < 0.001, ns represents *P* ≥ 0.05. **(B)** Pearson correlation assessing the relationship between HRGs and 28 types of immune cells.

We analyzed the differences in immune cell infiltration levels between cluster1 and cluster2 (**Figure 8A**). The results indicated that, compared to cluster2, cluster1 exhibited upregulated infiltration of CD56^dim^ natural killer cells, immature dendritic cells, T follicular helper cells, type 1 T helper cells, and type 17 T helper cells, and downregulated infiltration of eosinophils, gamma delta T cells, immature B cells, mast cells, and plasmacytoid dendritic cells. **Figure 8B** shows the correlation between HRGs and immune cells.

### LASSO analysis for selecting HBV-related genes

**Figure 9:**
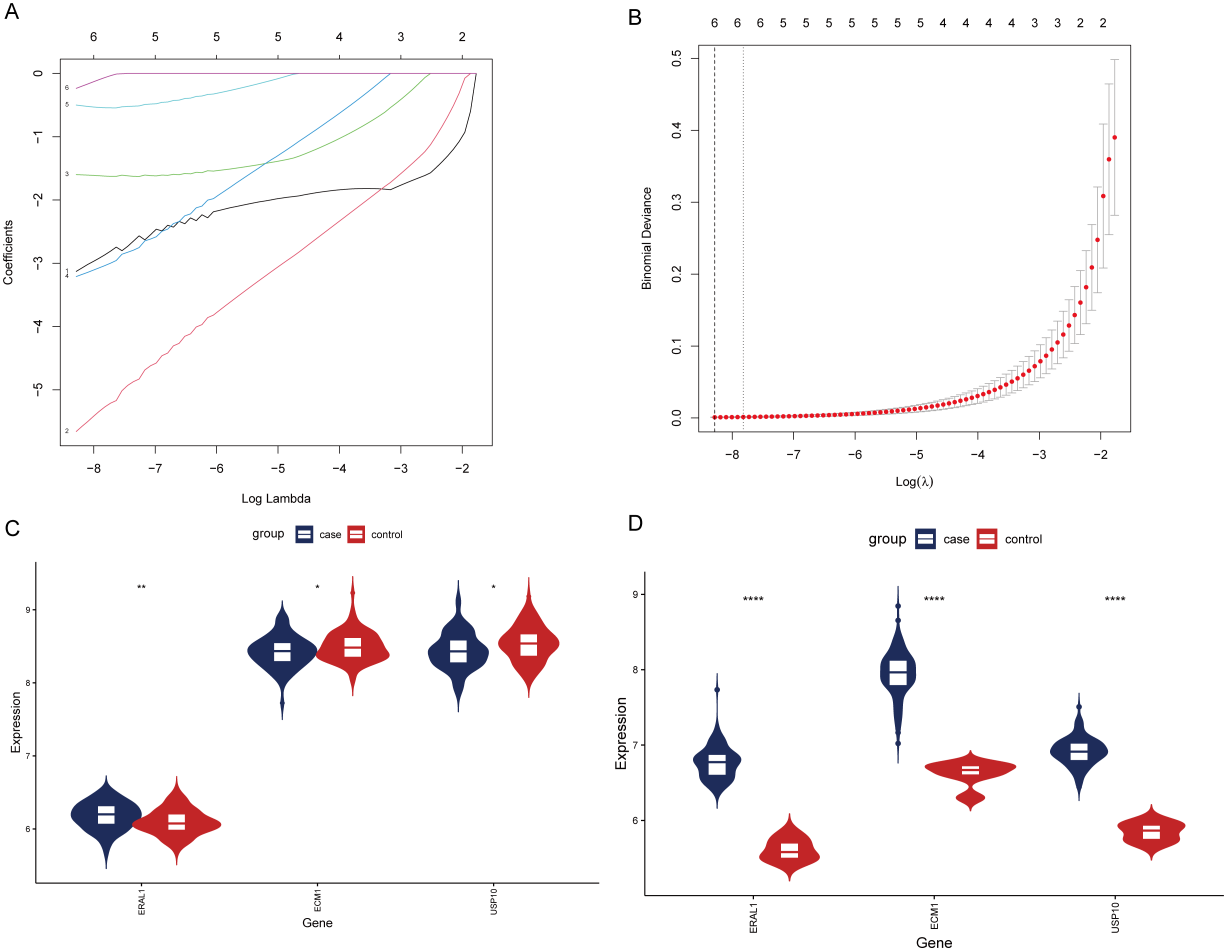
Screening of HRGs and expression of HRGs in the dataset. **(A)** Coefficients of each gene as the penalty parameter lambda varies. **(B)** Ten-fold cross-validation graph. **(C)** Expression of HRGs in the OP dataset. **(D)** Expression of HRGs in the HBV dataset. *P < 0.05, **P < 0.01, ****P < 0.0001.

After analyzing the OP dataset, we performed LASSO analysis on the GSE83148 dataset to further select HRGs **Figure 9A** illustrates the change in gene coefficients with lambda during the LASSO analysis. At lambda = 0.0002521665, where the Binomial Deviance was minimized, 6 genes were identified (**Figure 9B**). An intersection with HRGs yielded 3 genes: USP10, ERAL1, and ECM1. **Figures 9C, D** show the expression of these three genes in the OP and CBI datasets, respectively.

### The validation of the expression pattern of three hub genes

**Figure 10:**
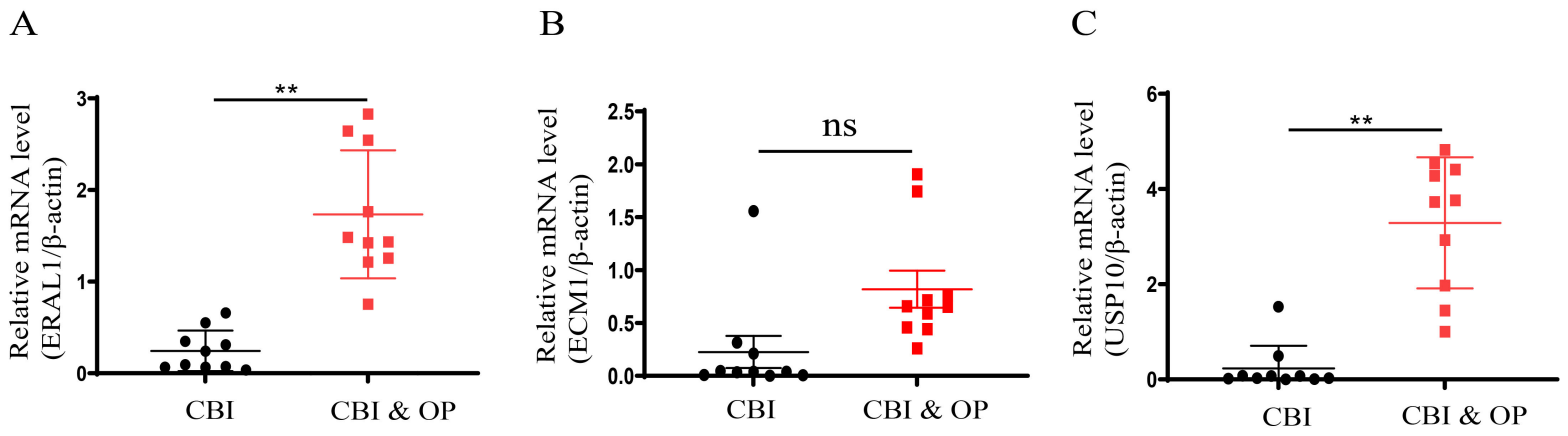
Expression of the three hub genes in CBI and CBI combined OP was detected by RT-qPCR. **(A)** ERAL1. **(B)** ECM1. **(C)** USP10. ns represents *P* ≥ 0.05, **P < 0.01.

To further confirm the accuracy of the above integrated bioinformatics analysis, we firstly examined the expression pattern of the three hub genes in the recruited patients. The RT-qPCR results confirmed expression pattern of three hub genes in CBI and CBI combined with OP. **Figure 10** shows the relative expression levels of the hub genes we identified in patients from the CBI group and the CBI combined with OP group. The relative expression levels of ERAL1 and USP10 in the CBI combined with OP group were significantly higher than those in the CBI group, consistent with the machine learning results (**Figures 10A, C**). Probably due to insufficient sample size, we did not observe differences in another hub gene ECM1 between the two groups (**Figure 10B**).

## Discussion

CBI is a global public health issue that not only significantly affects the liver but is also associated with a variety of non-hepatic complications, including OP (40, 41). OP is a systemic bone disease caused by multiple factors, leading to decreased bone density and quality, as well as the deterioration of bone microarchitecture (42). An increasing body of research suggests that CBI can elevate the risk of developing OP. Several studies have indicated that CBI may lead to osteoporosis through several mechanisms. Firstly, vitamin D deficiency, which is common in CBI patients, adversely affects bone metabolism and results in decreased bone mineral density. Additionally, long-term use of antiviral drugs, particularly tenofovir disoproxil fumarate (TDF), has been associated with bone mineral density reduction, likely due to its impact on renal phosphate handling, leading to bone demineralization. As CBI patients age, the presence of comorbidities such as diabetes, hypertension, and cardiovascular diseases further exacerbates the risk of osteoporosis by altering calcium metabolism and increasing bone fragility. Chronic inflammation associated with CBI may also contribute to bone loss by promoting the production of cytokines that enhance bone resorption. Although the direct effects of HBV on bone cells are still under investigation, these combined factors indicate that CBI patients, especially those on long-term TDF therapy and those with additional risk factors like age and comorbidities, are at a significantly increased risk of developing osteoporosis (43). Therefore, careful monitoring and individualized treatment strategies are recommended to mitigate this risk. Recently, the widespread adoption of microarray technologies and sequencing methods has significantly advanced the investigation of molecular mechanisms and landscapes of various diseases (44, 45). With the advent of big data, there is a growing utilization of comprehensive bioinformatics analysis and machine learning tools. These methods are instrumental in discovering new genes and potential diagnostic markers, as well as in unveiling the underlying mechanisms of diseases and identifying novel therapeutic targets (46, 47). This opens new avenues for understanding and treating diseases.

To our knowledge, this study is the first to explore the complex interactions between CBI and OP through the synergistic integration of bioinformatics analysis and machine learning techniques. In this research, we utilized datasets from the GEO database to identify HRGs by intersecting differentially expressed genes, conducting LASSO analysis, and employing machine learning recursive feature elimination. Notably, the HRGs selected by the three methods—RF-RFE, SVM-RFE, and GBM-RFE were consistent at 18, matching the LASSO analysis, indicating that HRGs are robust enough for predicting OP risk. Furthermore, the SVM model exhibited AUC values of 0.92, 0.83, 0.74, and 0.7 in the training set, validation set, GSE7429, and GSE7158, respectively. The nomogram model showed an AUC of 0.91 in the training set, with AUCs of 0.79 and 0.68 in the GSE7429 and GSE7158 datasets, respectively. Several OP prediction models based on biomarkers have been developed. The SVM prediction model constructed by Zhang Peng and others, based on m6A regulatory factors, achieved an AUC of 0.848 (48). The SVM prediction model constructed by Lai Jinzhi and colleagues, based on genes related to the Wnt pathway, achieved an AUC of 0.762, and the nomogram model also reached a high AUC of 0.7 (49). The prediction models developed by Zheng Zhenlong and his team showed AUCs ranging from 0.667 to 0.999 in the training set and from 0.603 to 0.662 in the test set (50). These results all emphasize the effectiveness of using machine learning to predict OP. Compared to other studies, our research, whether through the SVM based on HRGs or the nomogram model, demonstrates good predictive ability.

Patients with OP were divided into two distinct clusters using NMF clustering. Functional enrichment analysis further elucidated the different biological processes and pathways regulated in each cluster. These analyses revealed potential complex molecular mechanisms through identified upregulated and downregulated pathways, which may underlie the clinical manifestations of OP observed in patients with CBI. For instance, the differential regulation of pathways such as the Thyroid hormone signaling pathway, Th1 and Th2 cell differentiation, and the Adipocytokine signaling pathway in the GSEA results for KEGG highlighted the multifaceted nature of the pathogenesis of OP in the context of CBI (51–53). Additionally, pathways related to immune regulation, including Neutrophil extracellular trap formation, RIG-I-like receptor signaling pathway, Th1 and Th2 cell differentiation, FcγR-mediated phagocytosis, Adipocytokine signaling pathway, and B cell receptor signaling pathway, were upregulated in cluster1. Therefore, we speculate that cluster1 may predominantly influence the progression of OP through immune regulation. Conversely, in the GSVA results for KEGG, cluster2 exhibited upregulation in pathways such as primary bile acid biosynthesis, glycosaminoglycan biosynthesis - keratan sulfate, folate biosynthesis, and phenylalanine metabolism. Thus, we infer that cluster2 mainly affects the progression of OP through the synthesis and metabolism of condition-specific substances. These findings underscore the inherent heterogeneity of CBI combined with OP, indicating that the pathogenesis of OP in the context of CBI is not uniform but exhibits significant variation among individuals.

The analysis of immune cell infiltration has provided additional insights into the role of the immune system in the pathophysiology of chronic HBV-related OP. The differences in specific types of immune cell infiltrations between the two clusters emphasize the importance of the immune microenvironment in bone health and disease. The complex interactions between immune cells and bone cells, such as osteoblasts and osteoclasts, may influence bone density and structure, leading to the development or exacerbation of OP (54). Compared to cluster2, cluster1 exhibited increased infiltration of CD56^dim^ natural killer cells, immature dendritic cells, T follicular helper cells, Th1 cells, and Th17 cells, while showing decreased infiltration of eosinophils, γδT cells, immature B cells, mast cells, and plasmacytoid dendritic cells. These findings highlight the need for targeted therapeutic strategies that address both the viral infection and its immunological consequences to effectively manage chronic HBV-related OP.

Finally, the intersection of HRGs identified three genes: USP10, ERAL1, and ECM1. USP10 is an enzyme belonging to the ubiquitin-specific proteases (USPs) family, playing a key role in the de-ubiquitination process (55). De-ubiquitination refers to the removal of ubiquitin from ubiquitinated proteins, a post-translational modification that can signal protein degradation, alter protein location, affect activity, and promote or inhibit protein-protein interactions (56). The differentiation of mesenchymal stem cells into osteoblasts or the differentiation of monocytes into osteoclasts is regulated by USPs (57, 58). USP10 may participate in regulating bone metabolism processes through its specific de-ubiquitination activity, affecting the development of OP. Yu Wei and others found that estrogen can prevent cell aging and bone loss by regulating the degradation of p53 dependent on Usp10 in bone cells and osteoblasts (59). ERAL1 is an RNA chaperone located in mitochondria, mainly involved in the maturation and stability of mitochondrial 12s rRNA (60). ERAL1 is crucial for ensuring normal mitochondrial protein synthesis since 12s rRNA is a component of the mitochondrial ribosomal small subunit involved in protein synthesis within mitochondria (61, 62). Numerous studies have shown that mitochondrial dysfunction can lead to cellular disorder or dysfunction, disrupting the balance of osteoblast and osteoclast activity, thereby leading to the occurrence of OP (63, 64). Additionally, ERAL1 can promote the RIG-I-like receptor signaling pathway to inhibit viral infections (65). However, direct studies linking ERAL1 to OP have not been found. ECM1 is a widely expressed extracellular matrix protein that plays a role in various biological processes, including cell proliferation, differentiation, migration, and the organization and remodeling of the extracellular matrix (66–68). ECM1 influences the structure and function of the extracellular matrix through interactions with other extracellular matrix components such as collagens, glycoproteins, and proteoglycans (69). ECM can regulate the osteoblast lineage and osteoclast lineage, including their crosstalk, thereby affecting the occurrence of OP (70). It has been reported that Hepatocyte Growth Factor (HGF) and Epidermal Growth Factor (EGF) are increased in patients with HBV infection, enhancing the cell-protective intracellular signaling of ECM from the outside to the inside (71).

However, our study has some limitations. The research relied on datasets from publicly available data, with a limited number of samples and without specific datasets for CBI combined with OP, which may restrict the broad applicability of our findings. Furthermore, although we collected PBMCs from clinical patients and utilized qPCR to verify the expression levels of the hub genes for validation, the next step should involve collecting real-world data and detailed clinical information as supplements to verify the accuracy of the prediction models constructed with HRGs. Future studies should include additional experiments to explore the expression and mechanisms of USP10, ERAL1, and ECM in the context of CBI combined with OP. In clinical practice, particular attention should be given to patients with CBI exhibiting abnormal expression of USP10, ERAL1, and ECM, as this may indicate a higher risk of OP.

## Conclusion

In conclusion, this study successfully identified HRGs using a combination of bioinformatics analysis and machine learning. Furthermore, the SVM and nomogram models built based on HRGs demonstrated excellent predictive performance across various OP datasets. The HRGs divided OP patients into two HBV-related subgroups, which exhibited significant differences in immune cell infiltration and biological pathways.

## Data Availability

The raw data supporting the conclusions of this article will be made available by the authors, without undue reservation.
